# 

**Published:** 1881-02

**Authors:** 


### BOOKS AND PAMPHLETS RECEIVED.

How Persons Afflicted with Bright's Disease Ought to Live. By J. F. Edwards, M.D.

Lectures on Surgical Disorders of the Urinary Organs. By Reginald Harrison, F.R.C.S.

Manual of Medical Jurisprudence. By Alfred S. Taylor, M.D., F.R.S.

Transactions of State Medical Society of Pennsylvania, for 1880. Thirtieth Annual Session.

Yellow Fever: Its Ship Origin and Prevention. By R. B. S. Hargis, M.D., Pensacola, Florida.

Dr. Paul Börner's Reichs-Medicinal-Kalender für Deutschland, auf das Jahr 1880 und 1881.

Medical Heresies, Historically Considered; Evolution of Sectarian Medicine; Sketch and Review of Homœopathy. By Gonsalv C. Smythe, M.D.

Hernia, Strangulated and Reducible. By J. H. Warren, M.D.: Boston, Mass.

Compendium of Microscopical Technology. By Carl Seiler, M.D.

The Descriptive Atlas of Anatomy; A Representation of the Anatomy of the Human Body: In 92 Royal 4to Plates, Containing 550 figures. Philadelphia: J. B. Lippincott & Co. 1880.

Ophthalmic Test-Types, and Color Blindness Tests. Wm. Wood & Co., New York.

Atlas of Human Anatomy. Arranged according to Drs. Oesterreicher and Erdl; with Explanatory Text by J. A. Jeancon, M.D. A. C. Wilde & Co., Cincinnati. Complete in 45 parts, at 75 cents each. Numbers 983, 984, 985, 986, 987, 988, 989, 990, 991, and 992.

---

BY an error of the printers, the valuable selected article published by us last month, entitled "The Human Face," by Prof. Ambrose L. Ranney, of New York, was not duly accredited to the *New York Medical Journal.* We beg the indulgence of our esteemed cotemporary, as well as our many readers, for this apparent discourtesy. The *New York Medical Journal*, it need not be said, is one of the brightest and best of our exchanges, and we hope to go to it again for good selected material. We are sure that it will supply its readers with as good pabulum in the future as it has in the past.

DURING the last eighteen months, Prof. Wm. H. Byford, of Rush Medical College, has performed twenty-two ovariotomies with nineteen recoveries, a remarkably favorable showing, which the professor attributes, not so much to his well-known skill as an operator, as to the precautions taken in what is now well-known as Listerism.

SOCIETY MEETINGS.

Chicago Medical Society—Mondays, Feb. 7 and 21.
West Chicago Medical Society—Mondays, Feb. 4 and 28.
Biological Society—Wednesday, Feb. 2.

CLINICS.

MONDAY.

Eye and Ear Infirmary—2 p. m., Ophthalmological, by Prof. Holmes; 3 p. m., Otological, by Prof. Jones.

Mercy Hospital—2 p. m., Surgical, by Prof. Andrews.

Rush Medical College—2 p. m., Dermatological and Venereal, by Prof. Hyde.

Woman's Medical College—2 p. m., Dermatological and Venereal, by Prof. Maynard; 3 p. m., Diseases of the Chest, Prof. Ingals.

TUESDAY.

Cook County Hospital—2 to 4 p. m., Medical and Surgical Clinics.

Mercy Hospital—2 p. m., Medical, by Prof. Quine.

WEDNESDAY.

Chicago Medical College—2 p. m., Eye and Ear, by Prof. Jones.

Rush Medical College—2 p. m., Medical, by Dr. Bridge; 3 p. m., Ophthalmological and Otological, by Prof. Holmes; 3:30 to 4:30 p. m., Diseases of the Chest, by Dr. E. Fletcher Ingals.

THURSDAY.

Chicago Medical College—2 p. m., Gynæcological, by Prof. Jenks.

Rush Medical College—2 p. m., Diseases of Children, by Dr. Knox; 3 p. m., Diseases of the Nervous System, by Prof. Lyman.

Eye and Ear Infirmary—2 p. m., Ophthalmological, by Dr. Hotz.

Woman's Medical College—3 p. m., Surgical, by Prof. Owens.

FRIDAY.

Cook County Hospital—2 to 4 p. m., Medical and Surgical Clinics.

Mercy Hospital—2 p. m., Medical, by Prof. Davis.

SATURDAY.

Rush Medical College—2 p. m., Surgical, by Prof. Gunn; 3 p. m., Orthopœdic, by Prof. Owens.

Chicago Medical College—2 p. m., Surgical, by Prof. Isham; 3 p. m., Neurological, by Prof. Jewell.

Woman's Medical College—11 a. m., Ophthalmological, by Prof. Montgomery; 2 p. m., Gynæcological, by Prof. Fitch.

Daily Clinics, from 2 to 4 p. m., at the Central Free Dispensary, and at the South Side Dispensary.

